# Supplementary material for: Inferring the number of COVID-19 cases from recently reported deaths
Source: medRxiv. 2020 Mar 13:2020.03.10.20033761. Preprint. [Version 1] doi: 10.1101/2020.03.10.20033761 (PMC7239087; doi:10.1101/2020.03.10.20033761)
Supplement: Supplement 2020 [file 73470-2020.03.10.20033761-1.pdf]

Additional methodological information and complementary results

*This supplementary material is hosted by Eurosurveillance as supporting information alongside the article "**Inferring the number of COVID-19 cases from recently reported deaths**" on behalf of the authors who remain responsible for the accuracy and appropriateness of the content. The same standards for ethics, copyright, attributions and permissions as for the article apply. Eurosurveillance is not responsible for the maintenance of any links or email addresses provided therein."*

For details about the formatting of supplementary material please see our instructions for authors: https://www.eurosurveillance.org/for-authors

1. MODEL PARAMETERS

1.1. Serial interval

Our model requires some epidemiological parameters in order to simulate an epidemic of COVID-19. In order to ensure this epidemic reflects the true nature of the multiple global outbreaks of COVID-19, we draw from the most up-to-date probability distributions when such parameters are required.

One such parameter is the serial interval, which was taken from (1). The resulting distribution fitted is a Lognormal distribution with a mean of 4.7 days (95% CrI: 3.7, 6.0) and a SD of 2.9 days (95% CrI: 1.9, 4.9). Sampling and discretizing at the time scale of days gives the following distribution used in our analysis (Figure S1):

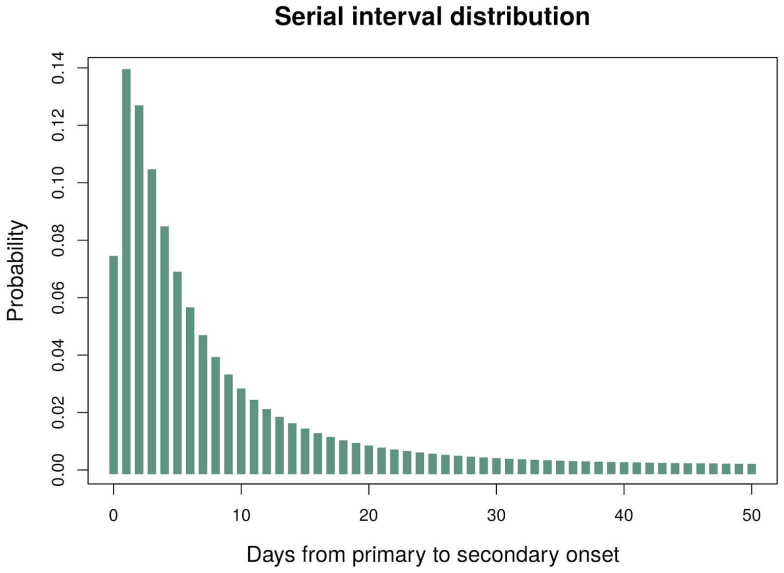

Figure S1: Probability mass function of the serial interval distribution.

1.2 Delay from onset to death

The distribution for the delay from onset-to-death was taken from Nishiura *et al.* (1), and implemented as a discretised Gamma distribution with a shape of 4.726 and a rate 0.3151, corresponding to a mean of 15 days and a standard deviation of 6.9 days.

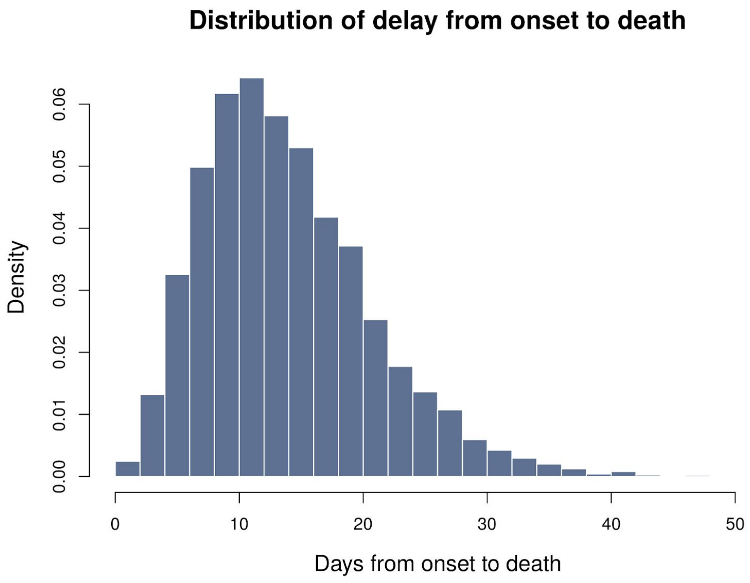

Figure S2: Probability mass function of onset-to-death distribution.

1.3 Number of cases per death

The numbers of cases per death was inferred from the case fatality ratio (CFR), as the number of Bernoulli trials (cases) of a Binomial distribution with 1 “success” (death) and a probability of success equal to the CFR. By definition, this means the number of cases can be drawn from a Geometric distribution with a probability equal to the CFR.

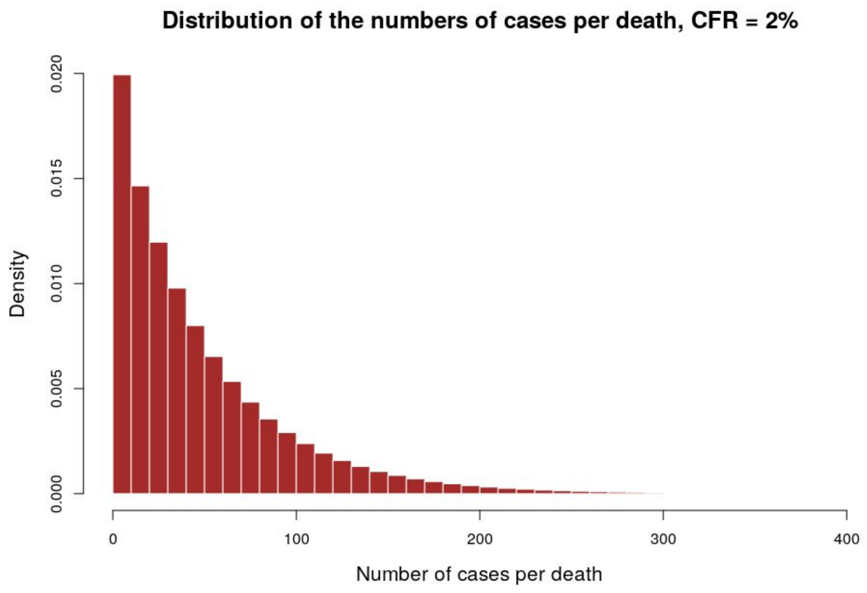

Figure S3: Probability mass function of the number of cases per death (CFR = 2%).

2. MODEL IMPLEMENTATION

Our model is implemented in a simulation algorithm involving the following steps:

For each death:

- draw a likely date of onset from the onset-to-death delay distribution; obtain one date of onset per death
- draw the total number of concurrent cases as the sample size of a Binomial distribution with probability = CFR and 1 success
- simulate several (50, by default) epidemic trajectories with a Poisson branching process using specified reproduction numbers (*R*) and serial interval distribution, until present time
- add epidemic trajectories from every deaths

A typical run of our model involves several hundreds (by default, 200) simulations, which are concatenated, and from which summaries are then derived.

All implementation has been realised in the R software (2), using the RECON packages *incidence* (3) and *projections* (4). Scripts and Rmarkdown documents implementing the model are freely available from github:
https://github.com/thibautjombart/covid19_cases_from_deaths/tree/master/analyses

A user-friendly, interactive web application implementing the model is available at:
https://cmmid.github.io/visualisations/inferring-covid19-cases-from-deaths

3. COUNTRY-SPECIFIC ANALYSES

3.1. Country

We obtained dates of deaths for countries with recently reported Covid-19 related deaths and no known ongoing transmission; United Kingdom, Spain, France, and Italy. Data was obtained from https://bnonews.com/index.php/2020/02/the-latest-coronavirus-cases/.

Results for 200 simulations are reported in the table below. We modelled different scenarios, using R of 1.5, 2, or 3, and CFR of 1, 2, 3, or 10%

country	R	CFR	reported	median	lower95	lower50	upper50	upper95
**Spain**	1.5	1 %	202	230	5	81	541	1 899
**Spain**	2	1 %	202	564	27	223	1 494	22 549
**Spain**	3	1 %	202	2 613	25	733	9 184	200 893
**Spain**	1.5	2 %	202	129	3	45	284	995
**Spain**	2	2 %	202	263	8	95	823	7 829
**Spain**	3	2 %	202	1 226	30	383	4 120	147 823
**Spain**	1.5	3 %	202	73	1	26	178	798
**Spain**	2	3 %	202	179	2	54	470	2 664
**Spain**	3	3 %	202	670	6	170	2 892	100 089
**Spain**	1.5	10 %	202	25	0	8	63	227
**Spain**	2	10 %	202	55	0	15	147	1 198
**Spain**	3	10 %	202	229	1	66	809	31 401
**Italy**	1.5	1 %	2037	1	596	20	219	1 596
**Italy**	2	1 %	2037	1	2 197	64	868	5 315
**Italy**	3	1 %	2037	1	24 298	570	8 245	115 267
**Italy**	1.5	2 %	2037	1	307	6	124	678
**Italy**	2	2 %	2037	1	1 087	15	284	2 644
**Italy**	3	2 %	2037	1	14 506	405	4 079	48 320

**Italy**	1.5	3 %	2037	1	194	5	91	392
**Italy**	2	3 %	2037	1	654	15	247	1 666
**Italy**	3	3 %	2037	1	9 512	198	3 143	31 839
**Italy**	1.5	10 %	2037	1	50	0	17	112
**Italy**	2	10 %	2037	1	224	1	81	606
**Italy**	3	10 %	2037	1	2 596	26	728	8 155
**France**	1.5	1 %	190	1	430	13	153	1 100
**France**	2	1 %	190	1	1 513	15	467	3 377
**France**	3	1 %	190	1	6 664	210	2 252	29 244
**France**	1.5	2 %	190	1	205	6	82	460
**France**	2	2 %	190	1	540	8	191	1 712
**France**	3	2 %	190	1	4 533	115	1 378	16 949
**France**	1.5	3 %	190	1	141	1	45	316
**France**	2	3 %	190	1	291	4	116	844
**France**	3	3 %	190	1	2 970	52	851	8 728
**France**	1.5	10 %	190	1	45	0	14	109
**France**	2	10 %	190	1	114	1	39	359
**France**	3	10 %	190	1	849	7	224	3 644

Table S1. Inferred number of cases at 4th of March in Spain, Italy, and France, assuming a net reproduction number (R) of 1.5, 2, and 3 and a case fatality ratio (CFR) of 1, 2, 3, and 10%.

4. NEGATIVE BINOMIAL ESTIMATES

We investigated the potential impact of heterogeneity in transmissibility using recent characterisation of the offspring distribution using a Negative binomial (k = 0.54 (5)). Results for a single death are reported in the table below.

R	cfr	median	lower_95	lower_50	upper_50	upper_95
1.5	0.01	261	6	97	618	2 714
2.0	0.01	640	7	244	1 763	8 908
3.0	0.01	2 989	35	555	11 177	188 220
1.5	0.02	121	5	49	311	1 894
2.0	0.02	289	5	79	758	6 023
3.0	0.02	1 358	8	310	4 190	52 341
1.5	0.03	75	2	29	240	776
2.0	0.03	183	2	58	517	2 313
3.0	0.03	853	9	227	3 425	66 083
1.5	0.10	27	0	9	56	205
2.0	0.10	56	0	14	184	1 228
3.0	0.10	204	3	53	644	12 055

Table S2. Impact of using a Negative binomial distribution for the number of secondary cases used in the branching process after introduction of a single death, on the date at which the death occurred. This table should be compared to table 1 in the main paper.

REFERENCES

1. Nishiura H, Linton NM, Akhmetzhanov AR. Serial interval of novel coronavirus (2019-nCoV) infections. medRxiv [Internet]. 2020; Available from: https://www.medrxiv.org/content/medrxiv/early/2020/02/17/2020.02.03.20019497.full.pdf

2. R Core Team. R: A Language and Environment for Statistical Computing [Internet]. Vienna, Austria: R Foundation for Statistical Computing; 2020. Available from: https://www.R-project.org/

3. Kamvar ZN, Cai J, Pulliam JRC, Schumacher J, Jombart T. Epidemic curves made easy using the R package *incidence*. F1000Res. 2019 Jan 31;8:139.

4. Project Future Case Incidence [R package projections version 0.3.1]. [cited 2020 Mar 4]; Available from: https://cran.r-project.org/web/packages/projections/index.html

5. Riou J, Althaus CL. Pattern of early human-to-human transmission of Wuhan 2019 novel coronavirus (2019-nCoV), December 2019 to January 2020. Euro Surveill [Internet]. 2020 Jan;25(4). Available from: http://dx.doi.org/10.2807/1560-7917.ES.2020.25.4.2000058
